# Tissue metabolite profiles for the characterisation of paediatric cerebellar tumours

**DOI:** 10.1038/s41598-018-30342-8

**Published:** 2018-08-10

**Authors:** Christopher D. Bennett, Sarah E. Kohe, Simrandip K. Gill, Nigel P. Davies, Martin Wilson, Lisa C. D. Storer, Timothy Ritzmann, Simon M. L. Paine, Ian S. Scott, Ina Nicklaus-Wollenteit, Daniel A. Tennant, Richard G. Grundy, Andrew C. Peet

**Affiliations:** 1 Institute of Cancer and Genomic Sciences, University of Birmingham, Birmingham, UK; 2 Birmingham Children’s Hospital, Birmingham, UK; 3 University Hospitals Birmingham, Birmingham, UK; 4 Bimingham University Imaging Centre (BUIC), School of Psychology, University of Birmingham, Birmingham, UK; 5 Childrens Brain Tumour Research Centre, University of Nottingham, Nottingham, UK; 6 Department of Neuropathology, Nottingham University Hospitals NHS Trust, Nottingham, UK; 7 Institute of Metabolism and Systems Research, University of Birmingham, Birmingham, UK

## Abstract

Paediatric brain tumors are becoming well characterized due to large genomic and epigenomic studies. Metabolomics is a powerful analytical approach aiding in the characterization of tumors. This study shows that common cerebellar tumors have metabolite profiles sufficiently different to build accurate, robust diagnostic classifiers, and that the metabolite profiles can be used to assess differences in metabolism between the tumors. Tissue metabolite profiles were obtained from cerebellar ependymoma (n = 18), medulloblastoma (n = 36), pilocytic astrocytoma (n = 24) and atypical teratoid/rhabdoid tumors (n = 5) samples using HR-MAS. Quantified metabolites accurately discriminated the tumors; classification accuracies were 94% for ependymoma and medulloblastoma and 92% for pilocytic astrocytoma. Using current intraoperative examination the diagnostic accuracy was 72% for ependymoma, 90% for medulloblastoma and 89% for pilocytic astrocytoma. Elevated *myo*-inositol was characteristic of ependymoma whilst high taurine, phosphocholine and glycine distinguished medulloblastoma. Glutamine, hypotaurine and N-acetylaspartate (NAA) were increased in pilocytic astrocytoma. High lipids, phosphocholine and glutathione were important for separating ATRTs from medulloblastomas. This study demonstrates the ability of metabolic profiling by HR-MAS on small biopsy tissue samples to characterize these tumors. Analysis of tissue metabolite profiles has advantages in terms of minimal tissue pre-processing, short data acquisition time giving the potential to be used as part of a rapid diagnostic work-up.

## Introduction

Paediatric tumors of the central nervous system (CNS) are the most common solid cancers diagnosed in children^1^. They are now the highest cause of cancer related deaths in this population, causing death in around a third of cases after 10 years^1^. In broad terms the treatment for these tumors is maximal safe resection followed by chemotherapy and/or radiotherapy as appropriate according to treatment protocols. Whilst there has been an improvement in survival over the past 40 years there have been fewer gains recently^2^ and there is a pressing need to elucidate the molecular pathology as well as identify biomarkers of diagnosis and prognosis of these tumors.

In children, approximately 50% of CNS tumors occur in the posterior fossa, with the cerebellum being the most common site. The three most common tumors arising in the cerebellum are pilocytic astrocytoma, medulloblastoma and ependymoma^1^. Other tumors can occur in the cerebellum, most notably atypical teratoid/rhabdoid tumors (ATRT)^3^. Paediatric brain tumors are becoming well characterized in terms of their genetic and epigenetic alterations and clinically relevant subgroups have been identified^4–7^. Metabolomics is a powerful characterizing feature of tumors that may have clinical value. *In vivo* magnetic resonance spectroscopy (MRS) has previously been used to characterize the metabolite profiles of paediatric cerebellar tumors, and reported differences in several metabolites^8^. However, *in vivo* MRS produces low resolution spectra where high concentration metabolites dominate those of a lower concentration and makes quantification of overlapping resonances difficult.

Highly detailed spectroscopic studies can be performed on surgically resected tissue using high-resolution magic-angle spinning magnetic resonance spectroscopy (HR-MAS). The combination of a stronger magnetic field, the ability to rapidly spin the sample at 54.7° with regard to the magnetic field and the proximity of the radiofrequency coils to the sample leads to greatly improved spectral resolution^9^ and accurate quantitation of low abundance metabolites not visible using MRS. HR-MAS has been used with great success to examine the metabolite profiles of a wide range of cancers including prostate^10^, breast^11^ and lung^12^ cancers, as well as brain tumors from children^13^ and adults^14^. Many metabolite concentrations measured by *in vivo* MRS have been shown to correlate with *ex vivo* HR-MAS^15^. Better characterization of paediatric brain tumor tissue metabolite profiles has the potential to be translated to *in vivo* MRS. Increased metabolite quantitative accuracy would provide clinicians with valuable diagnostic and prognostic information. Given that HR-MAS is able to rapidly characterize the metabolite profile of small surgically resected tissue samples without any complex preparation, it shows potential to aid the surgical management of patients undergoing surgery for cerebellar tumors in real time. The treatment protocol for each of the four tumors in this study are different, and so obtaining a real time diagnosis is crucial to a child’s treatment both in terms of maximizing surgical outcome and minimizing the delay between surgery and chemo- or radiotherapy, particularly for fast growing, aggressive tumors such as ATRTs. Therefore, the primary aim of this study is to examine the diagnostic ability of HR-MAS using biopsy tissue in a retrospective manner.

Metabolism is the result of expression of metabolic enzymes, which are altered by the tumorigenic drivers of the tumors^16,17^. The concentrations of the metabolites can provide an insight into how the metabolic pathways are altered, and identify potential targets to interfere with tumor metabolism. The secondary aim of this study is to identify metabolic pathways that are differentially activated between the three tumor types.

## Methods and Materials

### Acquisition of tissue

Frozen diagnostic cerebellar tumor tissue for 24 pilocytic astrocytomas, 36 medulloblastomas, 18 ependymomas and 5 atypical teratoid/rhabdoid tumors (ATRTs) were acquired from Birmingham Children’s Hospital (BCH) and the Children’s Cancer and Leukaemia Group (CCLG) tissue banks. This study has Research Ethical Committee approval (NRES East Midlands-Derby, 04/MRE04/41) and CCLG Biological Studies Committee approval. Informed consent was obtained from the patient or parent/legal guardian. All methods were carried out in accordance with relevant guidelines and regulations. All experimental protocols were approved by the University of Birmingham. Tissue was snap frozen in liquid nitrogen shortly after surgical resection, prior to tumor treatment and stored at −80 °C. The final diagnosis was made following full histopathological examination of the tissue including immunohistochemistry where required and reviewed and agreed upon by multi-disciplinary teams.

### HRMAS preparation

HR-MAS was performed at the Henry Wellcome Building Biomolecular NMR Facility at the University of Birmingham. Tissue was cut with a scalpel over dry ice to fit into a 12 μl or 50 μl zirconium rotor before being weighed. The internal standard 3-(Trimethylsilyl)propionic-2,2,3,3-d4 acid sodium salt (TMSP) (Cambridge Biosciences, Cambridge, UK) was added to the sample in a rotor dependent manner. 3 μl of standard was added to 12 μl rotors whilst 5 μl of standard was added to 50 μl rotors. D2O (Sigma Aldrich, Dorset, UK) was added to completely fill the rotor before it was fully assembled. The sample and rotor were kept cold over dry ice during preparation to prevent tissue degradation.

### Spectra acquisition

Spectra were acquired using a Bruker Avance spectrometer (Bruker, Coventry, UK) with a magnetic field strength of 500 MHz fitted with a 4 mm three channel HCD HRMAS z-PFG band probe. The rotor was spun at a temperature of 4 °C to prevent metabolic activity and a frequency of 4800 Hz to remove spinning sidebands from the spectra. A NOESY pulse and acquire sequence was used with 2 s presaturation to suppress the water signal and a repetition time of 4 s. 256 or 512 averages were acquired for 50 μl and 12 μl rotors respectively. Using this protocol, an experiment with 256 averages was completed in 17 minutes.

### Analysis

Free Induction Decays were Fourier transformed in Topsin 2.0 (Bruker, Coventry, UK) and the resulting spectra were imported into MestReNova 9.0.1 software suite (Mestrelab Research, Spain). To ensure quality control, the spectra were visually examined for a high signal to noise ratio, a well-defined TMSP peak and clear discrimination of the choline, phosphocholine and glycerophosphocholine peaks at 3.2–3.3 ppm. Based on *in vivo* ^1^H and ^31^P spectroscopy, normal tissue is expected to have a large NAA peak, small peaks corresponding to the choline containing compounds with a PCh/GPC ratio of 0.4, an intermediate creatine peak and very small lipid peaks^8,18^. Overall the metabolite profiles of these brain tumors differ greatly from normal brain tissue. The spectra were phased, baseline corrected and the chemical shift referenced with respect to TMSP at 0 ppm. Peaks were picked using the Global Spectral Deconvolution algorithm which improves the resolution of areas of the spectrum with overlapping resonances. Features were quantified by comparing the area of the peaks corresponding to the respective metabolite to the area of the internal standard peak, taking into account the number of protons contributing to both the metabolite and standard signal. Thirty one features were assigned (26 metabolites and 5 lipid groups; Supplementary Table 2) to the spectra and raw metabolite concentrations were obtained using the qNMR function in MestReNova. Experimentally acquired chemical shift information from Govindaraju *et al*.^19^ and the Human Metabolome Database (HMDB)^20^ were used to confirm signal assignment. Due to the low proportion of the tumors in which n-acetylaspartylglutamate and acetone could be confidently identified, and due to lactate accumulation in the tissue over a variable amount of time during surgical excision, these three metabolites were excluded from further analysis. Prior to statistical analyses, lipid concentrations were summed and the data were normalized to the sum of total non-lipid metabolite concentrations.

Normalized values were imported into the R statistical environment^21^ for analyses. The differences in normalized metabolite concentrations were tested for significance using a Kruskal-Wallis test, followed by post hoc Nemenyi tests. Bonferroni correction was applied to account for multiple comparisons. Each metabolite and total lipid was scaled to a mean of 0 and a standard deviation of 1 in order to perform principal component analysis and cluster analysis and visualize the structure of the data. Initially, a classification model was created for medulloblastoma, ependymoma and pilocytic astrocytoma cases. Principal components accounting for 90% of the total variation were then subjected to LDA. The robustness of the resulting model was assessed using a 10-fold cross validation. This technique builds the classifier on 90% of the cases selected randomly and tests it on the remaining 10% which therefore form an independent test set. The process is repeated 10 times so that all cases are included in a test set. A second model was created to incorporate the ATRT and to investigate the influence of adding rarer tumors to the analysis. Due to the low number of ATRT samples, a decision tree was used to classify samples as either embryonal or glial tumors, before attempting to classify the diagnosis. This model was validated using a leave-one-out cross validation, leading to each case being independently tested using a model created using the rest of the cohort.

Metabolic pathway analysis was conducted using the MetPA tool of Metaboanalyst 3.0^22^, an online server allowing network analysis of the tumors to be performed. MetPA conducts pathway enrichment analysis and pathway topology analysis to identify metabolic pathways that are differentially activated in the samples of interest.

The datasets generated and analysed during the current study are available from the corresponding author on reasonable request.

### Histopathological reports

BCH histopathological reports were available via the hospitals reporting system. The histopathological information for samples from other centres was requested through the CCLG tissue bank. For assessing the accuracy of current rapid intraoperative diagnosis, the rapid diagnosis was considered concordant if the diagnosis obtained via haematoxylin and eosin (H&E) stained smear preparations, frequently complemented by frozen sections, agreed with the final confirmed diagnosis for the case. Partial concordance was assigned when a distinct histopathological entity was diagnosed within a broader category, for example, an ependymoma described as a glial tumor.

## Results

### Cohort

In total, 83 samples were accrued, including 24 pilocytic astrocytomas, 18 ependymomas, 36 medulloblastomas and 5 ATRTs. The cohort features a male bias, in line with the fact that CNS tumors are known to be more prevalent in males^1^ (Supplementary Table 1). There is no statistical difference between the observed and expected numbers of males and females (χ^2^ p = 0.12).

### Metabolic profiles of paediatric cerebellar tumors differ by tumor type

**Figure 1:**
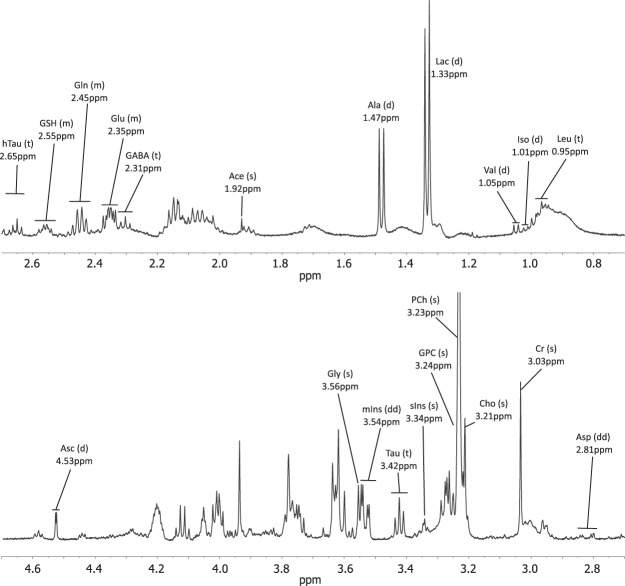
Example spectrum of a medulloblastoma acquired using HR-MAS. Metabolite assignments are shown along with their peak pattern. The resolution of the spectrum allows the accurate assignment and quantitation of a range of metabolites. Abbreviations: s - singlet, d – doublet, dd – doublet of doublets, t – triplet, m – multiplet, Ace – acetate, Ala – alanine, Asc – ascorbate, Asp – aspartate, Cho – Choline, Cr – Creatine, GABA - gamma-aminobutyric acid, Gln – glutamine, Glu – Glutamate, Gly – Glycine, GSH – glutathione, Htau – hypotaurine, Iso – Isoleucine, Leu – leucine, mIns - *myo*-inositol, sIns - *scyllo*-inositol, Tau – taurine, Val - valine.

The HR-MAS protocol allowed the acquisition of high resolution metabolic profiles (Fig. 1). Of the 25 quantified metabolites in the analysis, 14 were significantly different between the three tumor types (Table 1). Medulloblastoma displayed significantly higher concentrations of ascorbate, aspartate, phosphocholine, taurine and lipids at 1.3 ppm and significantly lower glucose and *scyllo*-inositol. Ependymomas showed significantly higher concentrations of *myo*-inositol and glutathione and significantly lower leucine. Pilocytic astrocytoma tumors had significantly higher concentrations of glutamine and hypotaurine. When medulloblastomas and ATRTs were compared, creatine was significantly lower in ATRTs (Supplementary Table 3).

**Table 1:** Kruskal Wallis tests identify 14 metabolites with concentrations that vary according to diagnosis.

Metabolite	Mean normalized concentration			Kruskall-Wallis P value	Bonferroni corrected P value	Nemenyi post hoc P values		
	Medulloblastoma	Ependymoma	Pilocytic astrocytoma			Med vs Epen	Med vs PA	Epen vs PA
Ascorbate	0.039	0.018	0.022	3.2 × 10^−5^	8.0 × 10^−4^	4.0 × 10^−4^	2.0 × 10^−3^	N.S
Aspartate	0.0076	0.0014	0.0009	2.8 × 10^−5^	7.0 × 10^−4^	8.6 × 10^−3^	1.0 × 10^−4^	N.S
Glucose	0.002	0.014	0.019	2.5 × 10^−4^	6.2 × 10^−3^	1.3 × 10^−2^	1.1 × 10^−3^	N.S
Glutamine	0.093	0.14	0.26	1.4 × 10^−9^	3.5 × 10^−8^	N.S	1.4 × 10^−9^	4.2 × 10^−3^
GPC	0.014	0.031	0.036	2.2 × 10^−5^	5.5 × 10^−4^	N.S	2.6 × 10^−5^	N.S
Glutathione	0.026	0.041	0.023	1.0 × 10^−4^	2.5 × 10^−3^	N.S	3.6 × 10^−2^	1.1 × 10^−4^
Hypotaurine	0.022	0.017	0.045	1.3 × 10^−4^	3.2 × 10^−3^	N.S	2.2 × 10^−2^	1.7 × 10^−4^
Leucine	0.0082	0.0014	0.0096	1.7 × 10^−4^	4.2 × 10^−3^	1.7 × 10^−4^	N.S	3.7 × 10^−2^
*Myo* inositol	0.12	0.28	0.13	2.0 × 10^−7^	5.0 × 10^−6^	6.5 × 10^−7^	N.S	4.1 × 10^−5^
NAA	0.013	0.0036	0.025	3.5 × 10^−7^	8.8 × 10^−6^	4.2 × 10^−3^	1.9 × 10^−2^	3.5 × 10^−7^
PCh	0.12	0.03	0.03	1.3 × 10^−10^	3.3 × 10^−9^	2.5 × 10^−6^	1.6 × 10^−8^	N.S
*Scyllo* inositol	0.0019	0.0072	0.008	1.2 × 10^−5^	3.0 × 10^−4^	2.3 × 10^−4^	8.0 × 10^−4^	N.S
Succinate	0.0024	0.0049	0.0099	7.7 × 10^−5^	1.9 × 10^−3^	N.S	7.9 × 10^−5^	N.S
Taurine	0.17	0.088	0.037	6.6 × 10^−11^	1.7 × 10^−9^	8.9 × 10^−3^	8.1 × 10^−11^	1.4 × 10^−2^

Post hoc tests identify which tumors have significantly different metabolite concentrations. Abbreviations – GPC, glycerophosphocholine; NAA, N-acetylaspartate; PCh, phosphocholine.

**Figure 2:**
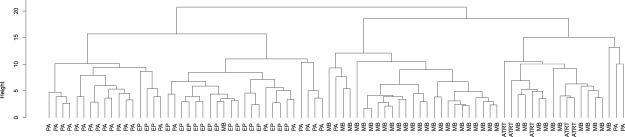
Hierarchical clustering of paediatric cerebellar tumors based on metabolite concentrations. The highest split in the figure separates glial tumors from embryonal tumors. The subsequent split broadly separates the tumor types. Abbreviations: MB, medulloblastoma; EP, ependymoma; PA, pilocytic astrocytoma; ATRT, Atypical Teratoid/Rhabdoid Tumor.

Unsupervised hierarchical clustering shows four distinct clusters (Fig. 2). The highest split in the dendrogram separates embryonal tumors from glial tumors. Each sub cluster largely corresponds with a particular tumor type. In particular, it is interesting to note that ATRT cases cluster together within one of the four sub clusters.

### Paediatric cerebellar tumors can be discriminated using linear discriminant analysis (LDA)

**Figure 3:**
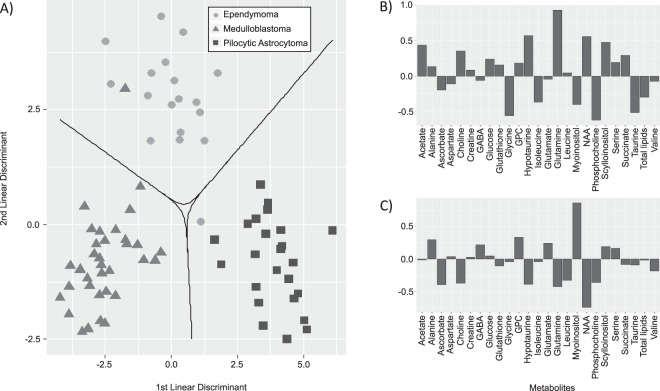
The output of the linear discriminant analysis. (**A**) The LDA scatterplot displays clear separation of the ependymoma (n = 18), medulloblastoma (n = 36) and pilocytic astrocytoma (n = 24). The decision boundaries, shown by the solid line, define the regions of the plot for each tumor type. (**B**) The loadings for the first discriminant function. The metabolites with more negative loadings are important for medulloblastoma classification, whilst metabolites with more positive loadings are important for pilocytic astrocytoma classification. (**C**) The loadings for the second discriminant function. Metabolites with more positive loadings are important for separating ependymoma from the other two tumor types. Abbreviations – GABA, gamma-aminobutyric acid; GPC, glycerophosphocholine; NAA, N-acetylaspartate; PCh, phosphocholine.

The LDA model shows clear separation of the three main tumor types (Fig. 3). The first linear discriminant function separates the medulloblastoma and pilocytic astrocytoma cases. Metabolites important for identifying medulloblastoma include phosphocholine, glycine, taurine and isoleucine. Glutamine, NAA, *scyllo*-inositol, hypotaurine and acetate are important for pilocytic astrocytoma classification. Ependymoma are separated from the other two tumor types by the second discriminant function. Metabolites important for ependymoma classification include *myo*-inositol, glycerophosphocholine, glucose and alanine.

The robustness of the model was tested using a 10-fold cross validation. Ependymoma and medulloblastoma had the joint highest classification accuracy with 94.4% of cases correctly classified. Pilocytic astrocytoma had the lowest accuracy with 91.7% of cases being classified correctly (Table 2). The high accuracy shows the model does not over fit the data and retains generalizability.

**Table 2:** The cross-validated diagnostic accuracy of the LDA for the three cerebellar tumor types.

Classification accuracy of linear discriminant analysis				
	Ependymoma	Medulloblastoma	Pilocytic astrocytoma	% correct
Ependymoma	17	0	1	94.4
Medulloblastoma	1	34	1	94.4
Pilocytic astrocytoma	1	1	22	91.7
**Rapid intraoperative histopathological diagnostic accuracy**				
	Number of concordant diagnoses	Number of partially concordant diagnoses	Number of discordant diagnoses	% concordant
Ependymoma	10	4	0	71.4
Medulloblastoma	28	1	2	90.3
Pilocytic astrocytoma	16	0	2	88.9

The linear discriminant classifier achieved accuracies greater than 90% for all three tumor types. Furthermore, for all tumor types, the classification accuracy was greater than the rapid intraoperative diagnostic examination; however, this appears to be due to partial concordance as opposed to an incorrect diagnosis.

Although this study has focused on the three most common childhood tumors presenting in the cerebellum, five ATRT tumors were added to see how tumor classification was affected and how accurately this minority tumor class could be classified. Using a binary classification model validated by a leave-one-out cross validation, classification accuracies of 60% for ATRT, 83.3% for medulloblastoma, 94.4% for ependymoma and 91.7% for pilocytic astrocytoma were obtained (Supplementary Fig. 1).

### Histopathological discrimination of paediatric cerebellar tumors

Ependymoma had the lowest diagnostic accuracy with 10 of the 14 cases (71%) reporting a concordant diagnosis where intra-operative preparations were assessed (Table 2). The remaining 4 cases were partially concordant; these samples were described as gliomas or low grade gliomas.

The pilocytic astrocytoma group were intermediate in the analysis, with 16 of 18 cases (89%) reporting a concordant diagnosis where an intra-operative diagnosis was given. The remaining two cases were discordant and were diagnosed as medulloblastoma and ependymoma.

Medulloblastoma had the highest rate of concordant diagnoses using rapid intraoperative preparations, with 28 of the 31 cases (90%). Two cases were discordant; one case was thought to represent an astrocytoma whilst the other a low grade glioma. The remaining case was partially concordant as it was diagnosed as a malignant intrinsic tumor. Of the 5 ATRT cases, 2 (40%) reported a concordant diagnosis. The remaining 3 were partially concordant; two were given with a medulloblastoma differential diagnosis and one was described as a high grade tumor.

The individual metabolite profile classification results of the partially concordant and discordant cases were reviewed. All partially concordant and discordant pilocytic astrocytoma, ependymoma and medulloblastoma were correctly classified by the LDA. Of the 3 partially concordant ATRT cases, 1 was correctly classified by the decision tree, whilst 1 was classified as an ependymoma and the other as a medulloblastoma.

### Metabolic pathways are altered between the three main tumor types

The normalized metabolite concentrations were submitted to the MetPA online tool^22^ for pathway analysis to identify altered pathways between the tumor groups. The four most statistically differentially activated pathways were: glycerophospholipid metabolism; taurine and hypotaurine metabolism; alanine, aspartate and glutamate metabolism; and arginine and proline metabolism (Table 3). The alanine, aspartate and glutamate had the highest impact factor, indicating that the key metabolites in this pathway were present in the analysis and that the tumors use these metabolites differently relative to each other. Conversely, arginine and proline metabolism had the lowest impact factor of the four pathways, indicating a lack of key metabolite quantification. Post hoc tests revealed that glycerophospholipid metabolism is enriched in medulloblastoma relative to the other two tumor types.

**Table 3:** The top 4 most significantly different pathways between the three most common cerebellar tumors.

Metabolic pathway	Total number of compounds	Hits	P value	Impact	Pairwise P values		
					Med vs Epen	Med vs PA	Epen vs PA
Glycerophospholipid metabolism	39	4	5.5 × 10^−20^	0.20	1.0 × 10^−8^	8.6 × 10^−11^	N.S.
Taurine and hypotaurine metabolism	20	4	3.6 × 10^−15^	0.44	1.9 × 10^−3^	9.1 × 10^−9^	4.9 × 10^−5^
Alanine, aspartate and glutamate metabolism	24	7	5.2 × 10^−15^	0.81	6.0 × 10^−3^	3.2 × 10^−11^	2.0 × 10^−4^
Arginine and proline metabolism	77	5	1.3 × 10^−14^	0.087	5.9 × 10^−3^	4.8 × 10^−11^	2.5 × 10^−4^

Glycerophospholipid metabolism is enriched in medulloblastoma compared to the other two tumor types. The alanine, aspartate and glutamate metabolism pathway has the highest topological metric, and it is likely that this pathway is strongly associated with tumor type.

## Discussion

This work has demonstrated that the metabolite profiles of *ex vivo* tumor tissue are different for the three major paediatric tumors of the cerebellum through unsupervised analysis, and are sufficiently different that robust diagnostic classifiers can be built with high rates of diagnostic accuracy. Also, we have shown that metabolic pathway analysis using quantified metabolites can identify pathways with altered activity between tumor types giving the potential for future targeted treatments. Given the ease of sample handling, the short data acquisition time and potential for automated analysis, the technique could find a role in aiding clinical diagnosis. Although not investigated for the samples in this work, HR-MAS is a non-destructive method and prior studies suggest that post analysis tissue is of suitable quality for subsequent histological and molecular analysis^23,24^. HR-MAS and *in vivo* MRS share the same technical principles, therefore tissue studies such as ours can be used to inform and refine non-invasive spectroscopic examination.

Alternative tissue based techniques have been proposed in the literature to aid the real time surgical management of patients. The alternative metabolomic technique, mass spectrometry, has been shown to have potential with regards to diagnosing pediatric brain tumors^25^. Unlike HR-MAS, the metabolites must be extracted from the tissue rendering it unavailable for further study. Raman spectroscopy and optical coherence tomography are two non-metabolomic techniques that have been shown to have intra-operative diagnostic potential for brain tumors^26,27^. Both are limited by the tissue penetrance they can achieve. Due to their intrinsic technical properties, none of the above three techniques can be translated to a non-invasive methodology.

The *ex vivo* spectroscopy provides further evidence for characteristic metabolite profiles of these tumor types. In particular, high phosphocholine and taurine in medulloblastoma, high glutamine in pilocytic astrocytoma and high *myo*-inositol in ependymoma tumors were important for tumor identification. Previous work^28^ has compared the metabolite profiles of paediatric cerebellar tumors and our studies agree on most of the key characteristic metabolite markers. Our study showed that *myo-*inositol is higher in concentration in ependymoma than medulloblastoma, agreeing with *in vivo* MRS but contrary to the findings reported in Cuellar-Baena *et al*.^28^ This is likely to be a consequence of the larger sample size in our study particularly for ependymoma. Furthermore, our study constructs an accurate and robust classifier with clinical utility and identifies metabolic pathways with different activity.

It has been shown that the most concentrated metabolites in brain tumors are quantifiable *in vivo* and correlate with the *ex vivo* spectroscopy^15^. Further technical development of *in vivo* MRS has shown that it is possible to accurately measure the concentration of metabolites with overlapping resonances^29–31^. Therefore, the metabolites described in this work have the potential be to be used as diagnostic markers.

The decision boundaries clearly separate the tumor types, with only two cases lying outside the decision boundary for their respective tumor type. It is interesting to note the loadings of the discriminant analysis reflect the findings of the average concentrations of the metabolites discussed previously, confirming that typical spectroscopic features can be used to separate the three tumor types. The classification algorithm is robust against overfitting, as demonstrated by the high classification accuracy after 10-fold cross validation.

Whilst our preliminary results for ATRT classification are encouraging, incorporation of rare tumor types may require statistical oversampling methods to compensate for the large differences in tumor frequency^32^. ATRT are rare but important tumors in childhood; they represent the majority of the tumors in the cerebellum which are not medulloblastomas, pilocytic astrocytomas or ependymomas and commonly represent a diagnostic dilemma particularly with medulloblastoma^33^. They are particularly aggressive tumors with a very poor prognosis and for which it is essential that maximal resection is obtained and that adjuvant treatment is started as soon as possible. There is currently no rapid diagnostic test for this tumor group since diagnosis relies on immunohistochemical staining for INI1. On an interesting note, the classification accuracy for pilocytic astrocytoma and ependymoma remain the same when ATRTs are incorporated. Therefore, metabolite profiles are able to confidently identify and separate glial and embryonal tumors, which is important if the tumor in question is a rare entity.

HR-MAS has shown promise for examining tissue from several cancer types^10,11^, and may be used to complement the histological data. Indeed, some work has been published on the use of rapid metabolite profiling and its clinical application in the surgical management of tumors^34^ which reported histopathology matching tumor identification in 17 mins. With metabolism under the control of the oncogenic drivers, HR-MAS has the potential to identify tumor subgroups in a short time frame, as shown in adults with SDH mutated paraganglioma or pheochromocytoma^35^ or IDH mutated gliomas^36^. HR-MAS also has the ability to identify novel metabolic subgroups which correlate with histopathological features. This was demonstrated in retinoblastoma where 3 clinicopathological subgroups were identified in which taurine, lipids and phosphosholine correlated with differentiation, necrosis and invasion respectively^37^. Other work performed using neuroblastoma tissue found taurine correlated with a worse prognosis whilst glutamine, *myo*-inositol and glycine was associated with stage I-II neuroblastoma^38^. Whilst our study has not attempted to classify tumors into recognized molecular subgroups, there is evidence that MRS is able to identify medulloblastoma molecular subgroup^39^ and this warrants further investigation. Brain tumors in adults are different in terms of the types of tumors and their anatomical location^40^. HR-MAS has been used to predict grade of astrocytic tumors^41^, and assess the extent of microheterogeneity in glioblastoma^23^.

To put the accuracy of the HR-MAS classification into context, a comparison was made with accuracy of current rapid intraoperative assessment using clinical reports for the tissue used in this analysis. Rapid intraoperative testing consists of smear cytological preparations often complemented by frozen sections stained with H&E to assess the cytological aspect as well as architectural features^42^. This method is cheap, rapid without the need for expensive equipment and gives reasonably accurate results to guide surgical management decisions.

The results of our analysis broadly agree with published retrospective analyses of rapid intraoperative assessment^43,44^; complete agreement of the rapid intraoperative assessment and final histological diagnosis is achieved in the majority of astrocytoma and medulloblastoma cases. Ependymoma, however, have a higher rate of partial concordance. Currently, the greatest prognostic marker for ependymoma is extent of surgical resection^45^, therefore it is imperative to identify ependymomas and optimize surgical treatment. Although there were no discordant ependymoma diagnoses by intraoperative assessment in our study, the partially concordant diagnoses could include tumors for which a complete resection is far less important. HR-MAS may aid in the identification of such tumors in real time and improve surgical management when intraoperative H&E assessment is ambiguous. In support of this, the metabolite profiles of the partially concordant and discordant pilocytic astrocytoma, ependymoma and medulloblastoma were not different from those of their respective tumor types. Metabolite profiles aided in the identification of one of the partially concordant ATRT cases, but not in the other two.

The pathway analysis identified metabolic pathways that were differentially activated between the three tumor types. Of these pathways, the alanine, glutamate and aspartate metabolic pathway had the highest impact factor. Therefore, it is likely that the biochemistry within this pathway is different between tumor groups. This pathway is closely linked to supplementing the tricarboxylic acid cycle through the conversion of amino acids to TCA cycle intermediaries^46^ and provides nitrogen for the de novo nucleotide synthesis^47^. Upregulation of glutamine catabolising pathways has been linked to more aggressive, higher grade tumors which rely on glutaminolysis to support their increased proliferation^48^. Consequently, lower glutamine is observed in medulloblastoma and ependymoma compared to pilocytic astrocytoma.

Hypotaurine and taurine metabolism and glycerophospholipid metabolism also differed between the tumor types. The high concentration of taurine in embryonal tumors is likely related to their developmental stage when compared to the glial ependymoma and pilocytic astrocytoma, as high taurine concentration has been described in other paediatric embryonal CNS tumors^37,49^. Taurine has been shown to be important for neuronal stem cell proliferation and neurogenesis^50,51^ and the migration of neuronal cells from the external to the internal granular layer in the cerebellum^52^. Hypotaurine is a poorly understood metabolite whose function is not clear and warrants further study to elucidate its role in normal and neoplastic tissue.

Glycerophospholipid metabolism is primarily responsible for synthesizing components for cellular and organelle membranes. Alterations in choline metabolism is a hallmark of malignancies^53^. In brain tumors, increased concentration of choline containing compounds as detected by *in vivo* ^1^H MRS have been associated with higher grade^54^. In addition, ^31^P MRS has identified alterations in the ratios of phosphorylated choline metabolites between normal tissue and brain tumors^18^. This is supported by *ex vivo* work, where it was shown that phosphocholine concentration correlated with grade, phospholipase C and choline kinase β expression^55^. These factors influencing synthesis may explain the observed increase in phosphocholine concentration in medulloblastoma tissue. As well as meeting the cells biosynthetic needs, this pathway is involved in intracellular signalling. The action of phospholipase C enzymes generates diacylglycerol and a free head group such as phosphocholine or phosphoinositol which act as secondary messengers. Our results suggest this pathway is enriched in medulloblastoma, and is an ideal candidate pathway for further examination to identify targeted therapies.

The arginine and proline metabolism pathway was detected to be significantly different between the three tumor types. HR-MAS is not able to identify the very low abundance key metabolites in this pathway, leading to a low impact score and an inability to draw firm conclusions regarding this pathway. Further examination of the metabolic pathways using other more sensitive metabolomic approaches will improve the metabolome coverage and lead to a more comprehensive metabolic pathway analysis. This approach will be vital to gaining a complete understanding of how metabolism varies between these tumor types and identify potential treatment targets.

In conclusion, this study has shown that HR-MAS can detect characteristic metabolic profiles from small pieces of fresh frozen biopsy tissue which can be used to build an accurate and clinically relevant classifier, with potential for future development as an intraoperative technique for improved surgical management. It has also shown that biological information can be extracted and differentially activated metabolic pathways can start to be identified. This may lead to improved insights into the metabolism of these tumors and the development of targeted therapies.

## Electronic supplementary material

Supplementary information
