# Supplementary material for: Tissue metabolite profiles for the characterisation of paediatric cerebellar tumours
Source: Sci Rep. 2018 Aug 10;8:11992. doi: 10.1038/s41598-018-30342-8 (PMC6086878; doi:10.1038/s41598-018-30342-8)
Supplement: Supplementary file 1 — Supplementary information [file 41598_2018_30342_MOESM1_ESM.docx]

**Title: Tissue metabolite profiles for the characterisation of paediatric cerebellar tumours**

Christopher D. Bennett^1,2^, Sarah E. Kohe^1,2^, Simrandip K. Gill^1,2^, Nigel P. Davies^2,3^, Martin Wilson^4^, Lisa C.D. Storer^5^, Timothy Ritzmann^5^, Simon M.L. Paine^6^, Ian S. Scott^6^, Ina Nicklaus-Wollenteit^2^, Daniel A. Tennant^7^, Richard G. Grundy^5^ and Andrew C. Peet^1,2*^

^1^Institute of Cancer and Genomic Sciences, University of Birmingham, Birmingham, UK, ^2^Birmingham Children’s Hospital, Birmingham, UK, ^3^University Hospitals Birmingham, Birmingham, UK, ^4^Bimingham University Imaging Centre (BUIC), School of Psychology, University of Birmingham, Birmingham, UK, ^5^Childrens Brain Tumour Research Centre, University of Nottingham, Nottingham, UK, ^6^Department of Neuropathology, Nottingham University Hospitals NHS Trust, Nottingham, UK, ^7^Institute of Metabolism and Systems Research, University of Birmingham, Birmingham, UK

**Supplementary information**

**Supplementary table 1** - Cohort demographics for cases used in the analysis.

| Diagnosis | WHO grade | N | Age at diagnosis (months) | | Gender | |
| --- | --- | --- | --- | --- | --- | --- |
|  |  |  | Median | Range | M | F |
| Pilocytic astrocytoma | I | 24 | 120 | 14-190 | 11 | 13 |
| Ependymoma | II/III | 18 | 32 | 14-194 | 13 | 5 |
| Medulloblastoma | IV | 36 | 69 | 18-210 | 27 | 9 |
| Atypical Teratoid Rhabdoid Tumour | IV | 5 | 2 | 1 - 55 | 3 | 2 |

**Supplementary table 2** - The ppm location and peak pattern for assigned metabolites.

| **Metabolite** | **^1^H chemical shift (ppm)** | **Multiplicity** |
| --- | --- | --- |
| Lipid | 0.9 | Broad singlet |
| Leucine (Leu) | 0.95 | Triplet |
| Isoleucine (Iso) | 1.01 | Doublet |
| Valine (Val) | 1.04 | Doublet |
| Lipid | 1.3 | Broad singlet |
| Lactate (Lac) | 1.33 | Doublet |
| Alanine (Ala) | 1.47 | Doublet |
| Lipid | 1.6 | Broad singlet |
| Acetate (Ace) | 1.92 | Singlet |
| N-acetylaspartate (NAA) | 2.02 | Singlet |
| N-acetylaspartylglutamate (NAAG) | 2.03 | Singlet |
| Acetone | 2.23 | Singlet |
| γ-amino butyric acid (GABA) | 2.30 | Triplet |
| Glutamate (Glu) | 2.35 | Multiplet |
| Succinate (Suc) | 2.41 | Singlet |
| Glutamine (Gln) | 2.45 | Multiplet |
| Glutathione (GSH) | 2.55 | Multiplet |
| Hypotaurine (hTau) | 2.65 | Triplet |
| Lipid | 2.8 | Broad singlet |
| Aspartate (Asp) | 2.82 | Doublet of doublets |
| Creatine (Cr) | 3.03 | Singlet |
| Choline (Cho) | 3.20 | Singlet |
| Phosphocholine (PCh) | 3.22 | Singlet |
| Glycerophosphocholine (GPC) | 3.23 | Singlet |
| Scyllo-inositol (sIns) | 3.34 | Singlet |
| Taurine (Tau) | 3.42 | Triplet |
| Myo-inositol (mIns) | 3.53 | Doublet of doublets |
| Glycine (Gly) | 3.56 | Singlet |
| Serine (Ser) | 3.84 | Doublet of doublets |
| Ascorbate (Asc) | 4.52 | Doublet |
| β D-glucose (Glc) | 4.65 | Doublet |
| Lipid | 5.3 | Broad singlet |

**Supplementary table 3 -** Comparison of normalized ATRT and medulloblastoma metabolite concentrations.

| **Metabolite** | **ATRT** | | **Medulloblastoma** | | **Kruskal-Wallis P value** | **Bonferroni adjusted P value** |
| --- | --- | --- | --- | --- | --- | --- |
|  | **Mean** | **SD** | **Mean** | **SD** |  |  |
| Acetate | 0.0051 | 0.0014 | 0.0038 | 0.0032 | 0.035 | 0.59 |
| Alanine | 0.070 | 0.041 | 0.049 | 0.036 | 0.18 | 1 |
| Ascorbate | 0.025 | 0.0083 | 0.039 | 0.024 | 0.13 | 0.97 |
| Aspartate | 0.0098 | 0.0096 | 0.0076 | 0.012 | 0.63 | 1 |
| Choline | 0.018 | 0.0069 | 0.017 | 0.016 | 0.22 | 1 |
| Creatine | 0.032 | 0.0089 | 0.094 | 0.034 | 0.0011 | 0.027 |
| GABA | 0 | 0 | 0.0057 | 0.018 | 0.33 | 1 |
| Glucose | 0 | 0 | 0.0020 | 0.0057 | 0.29 | 1 |
| Glutamate | 0.14 | 0.057 | 0.069 | 0.042 | 0.0076 | 0.17 |
| Glutamine | 0.074 | 0.01 | 0.093 | 0.039 | 0.32 | 1 |
| Glycine | 0.067 | 0.016 | 0.097 | 0.049 | 0.14 | 0.98 |
| GPC | 0.013 | 0.018 | 0.014 | 0.015 | 0.68 | 1 |
| Glutathione | 0.052 | 0.026 | 0.026 | 0.020 | 0.055 | 0.76 |
| Hypotaurine | 0.012 | 0.011 | 0.022 | 0.014 | 0.16 | 0.99 |
| Isoleucine | 0.0049 | 0.0016 | 0.0033 | 0.0037 | 0.042 | 0.66 |
| Leucine | 0.014 | 0.0096 | 0.0082 | 0.0075 | 0.17 | 0.99 |
| *Myo* inositol | 0.078 | 0.086 | 0.12 | 0.076 | 0.10 | 0.93 |
| NAA | 0.016 | 0.017 | 0.013 | 0.010 | 0.87 | 1 |
| Phosphocholine | 0.18 | 0.15 | 0.12 | 0.056 | 0.45 | 1 |
| Serine | 0.0082 | 0.012 | 0.0096 | 0.013 | 0.90 | 1 |
| Succinate | 0.0033 | 0.0014 | 0.0024 | 0.0022 | 0.22 | 1 |
| *Scyllo* inositol | 0.0021 | 0.0028 | 0.0019 | 0.0034 | 0.83 | 1 |
| Taurine | 0.16 | 0.063 | 0.17 | 0.093 | 0.97 | 1 |
| Valine | 0.016 | 0.0054 | 0.01 | 0.0094 | 0.0086 | 0.19 |
| Total lipids | 3.43 | 3.72 | 0.87 | 0.77 | 0.023 | 0.44 |

**Supplementary figure 1** – The decision tree for classifying pilocytic astrocytomas, ependymomas, medulloblastomas and ATRTs using metabolite profiles. The loadings at each level display how metabolite concentrations affect the classification of tumours. The first decision is to classify the tumour as a glial or embryonal tumour, before assigning a diagnosis at the second level.
